# Supplementary material for: The Iron Maiden. Cytosolic Aconitase/IRP1 Conformational Transition in the Regulation of Ferritin Translation and Iron Hemostasis
Source: Biomolecules. 2021 Sep 9;11(9):1329. doi: 10.3390/biom11091329 (PMC8469783; doi:10.3390/biom11091329)
Supplement: Supplementary file 1 [file biomolecules-11-01329-s001.zip › Supplementary/SI_ironmaiden.pdf]

Supplementary Information for:

The iron maiden. Cytosolic aconitase/IRP1 conformational transition in the regulation of ferritin translation and iron hemostasis

Cécilia Hognon[1], Emmanuelle Bignon[1], Guillaume Harle[2], Nadège Touche[2], Stéphanie Grandemange[2,*] and Antonio Monari[1,3*]

[1]Université de Lorraine and CNRS, UMR 7019 LPCT, F-5400 Nancy, France
[2]Université de Lorraine and CNRS, UMR 7039 CRAN, F-54000 Nancy, France
[3]Université de Paris and CNRS, ITODYS, F-75006 Paris, France.

[*]Correspondence: SG stephanie.grandemange@univ-lorraine.fr, AM Antonio.monari@univ-lorraine.fr

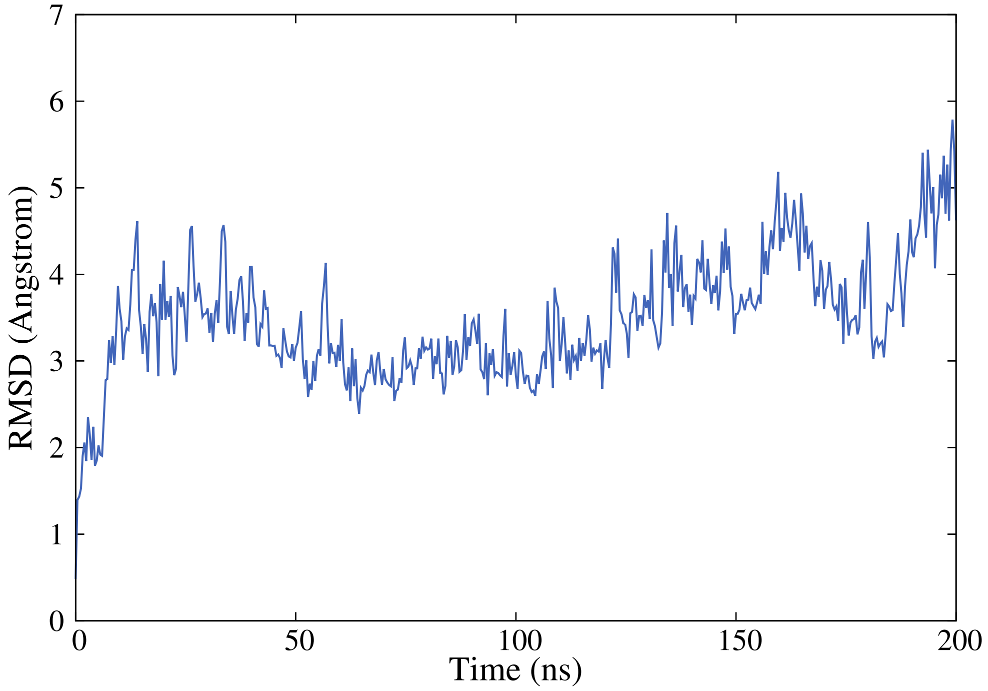

Figure S1. Time series of the RMSD for the isolated IRP1.

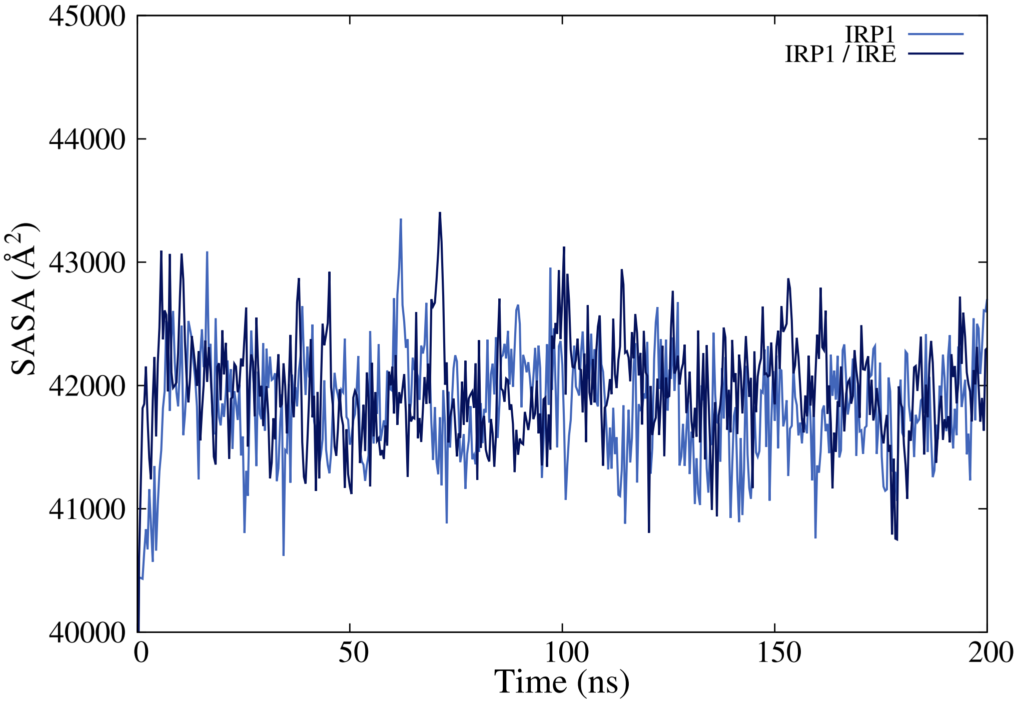

Figure S2. Time series of the Solvent Accessible Solvent Area (SASA) for isolated IRP1 and the IRE/IRP1 complex.

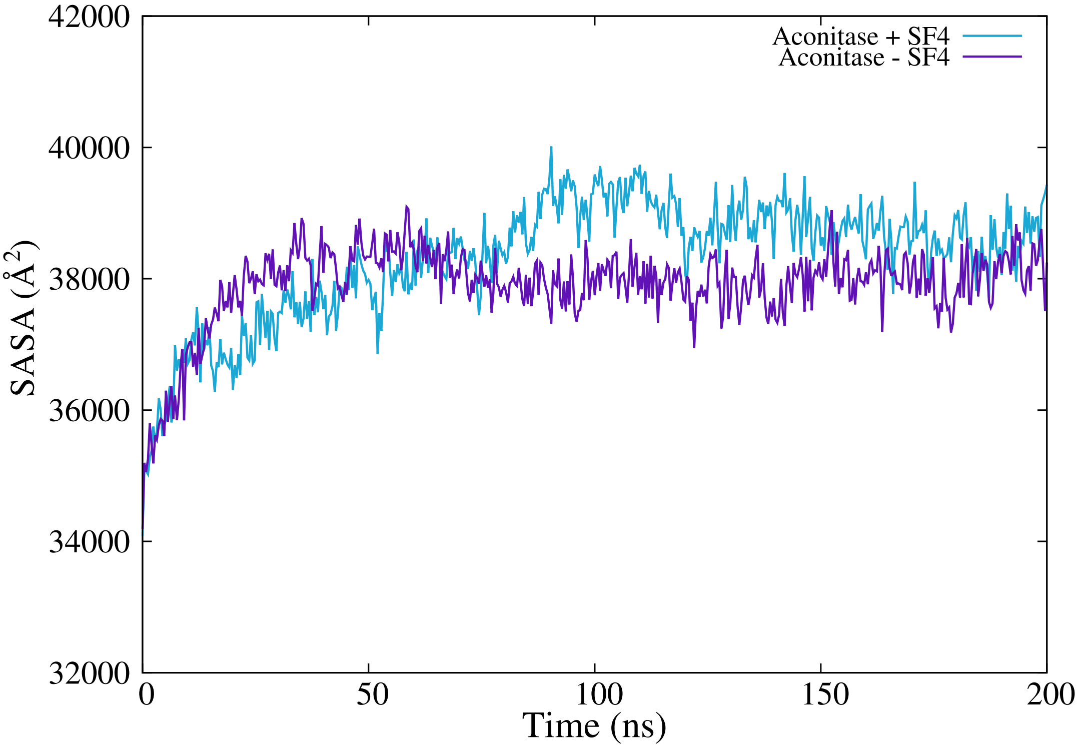

Figure S3. Time series of the Solvent Accessible Solvent Area (SASA) for cAco with and without the inclusion of the Fe-S cluster.

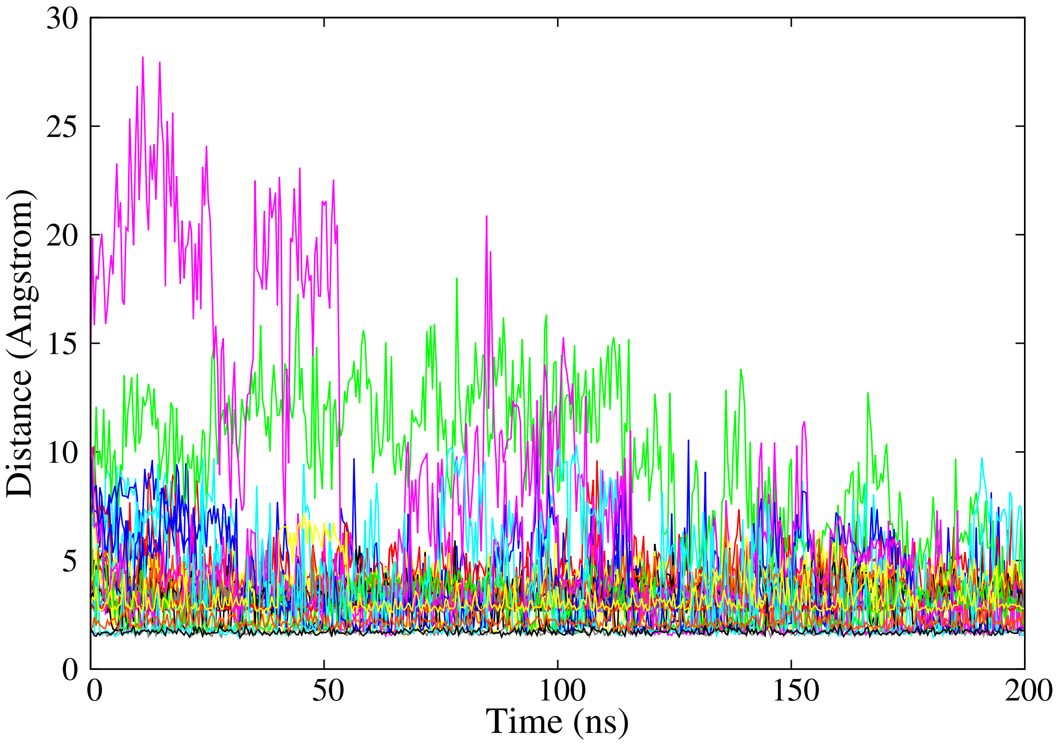

Figure S4. Time series of the distances between the interfacial residues of IRP1 and IRE.

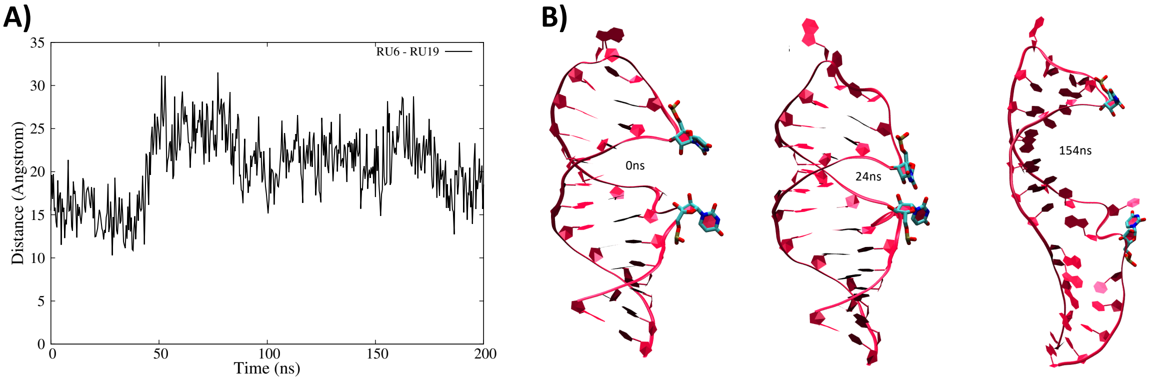

Figure S5. Time series of the distance between the center of mass of the two dangling uracil nucleobases (A) and representative snapshots extracted from the MD simulation (B).
